# Structural change of thalamus in cirrhotic patients with or without minimal hepatic encephalopathy and the relationship between thalamus volume and clinical indexes related to cirrhosis

**DOI:** 10.1016/j.nicl.2018.09.015

**Published:** 2018-09-20

**Authors:** Chun-Qiang Lu, Yun Jiao, Xiang-Pan Meng, Yu Cai, Ying Luan, Xiao-Min Xu, Shenghong Ju

**Affiliations:** Jiangsu Key Laboratory of Molecular and Functional Imaging, Department of Radiology, Zhongda Hospital, Medical School of Southeast University, Nanjing 210009, China

**Keywords:** VBM, Voxel-based morphometry, HE, Hepatic Encephalopathy, MHE, Minimal Hepatic Encephalopathy, NCT-A, Number Connection Test A, NCT-B, Number Connection Test B, DST, Digit Symbol Test, BDT, Block Design Test, MNI, Montreal Neurological Institute, GM, Grey Matter, WM, White Matter, CSF, Cerebrospinal Fluid, AAL, Automated Anatomical Labeling, VBM, Hepatic Encephalopathy, Grey Matter volume, Thalamus

## Abstract

Aberrant brain structural change in cirrhotic patients with or without hepatic encephalopathy is one of the most typical cases in voxel-based morphometry (VBM) studies. However, there exist inconsistent results regarding to the volume change of the thalamus. Furthermore, the relationship between thalamus structural change and cirrhotic symptoms has not yet been fully elucidated. To address these two issues, we repeated two VBM analyses in SPM and FreeSurfer and compared the two measurements with manually measured thalamic volumes. We also correlated the VBM results with clinical indexes related to cirrhosis to further investigate the relationship between thalamic structural change and liver cirrhosis. The inconsistent result of thalamic structural change was successfully reproduced in regard to the volume measurements of SPM and FreeSurfer. The manually measured results demonstrate an increase in the volume of the thalamus in cirrhotic patients compared to healthy controls, which differs from the results of FreeSurfer. The structural change of thalamus closely correlated with the blood biochemical indexes, including albumin levels, blood coagulation time, and AST/ALT ratio. All of these biochemical indexes are closely related to the severity of liver cirrhosis. Beyond all the results, this study also provides a good demonstration of the difference between multiple VBM measurements for clinicians.

## Highlights

• The inconsistent results of thalamus structural change are software dependent.
• The cirrhotic patients with hepatic decompensation have a larger thalamus volume than healthy controls.
• The grey matter volume of thalamus closely correlate with the blood biochemical tests related to liver cirrhosis.
• Relative intensity change is more sensitive than volume change in thalamus for discriminating the degree of cirrhosis.

## 1 Introduction

Voxel-based morphometry (VBM) is a neuroimaging technique that allows for the investigation of focal structural differences in the brain, and it has been widely used to characterize the patterns of brain structural changes in various neurodegenerative diseases (Ashburner and Friston, 2000; Good et al., 2001; Nickson et al., 2016; Voets et al., 2008; Yang et al., 2016). It is also a common method utilized to characterize brain structural differences regarding to different populations, such as difference in gender or ethnic (Ashburner et al., 1998; Chen et al., 2007; Ruigrok et al., 2014). Compared to other neuroimaging methods like fMRI, most of the VBM studies share relatively consistent results for a specific disease, such as Alzheimer's disease or drug abuse (Ersche et al., 2013; Matsuda, 2016). However, there also are some exceptions when different methodological choices were taken into account (Eckert et al., 2006; Ridgway et al., 2008). When reviewing past articles, we found there exists a discrepancy in the VBM results of thalamus in the cirrhotic patients (Chen et al., 2012; Montoliu et al., 2012; Tao et al., 2013; Zhang et al., 2012). In the study of Montoliu et al., the volume of thalamus is decreased in the cirrhotic patients with or without minimal hepatic encephalopathy (MHE) (Montoliu et al., 2012). However, according to the results of Zhang et al., Chen et al., and Tao et al., the thalamus of cirrhotic patients showed an increased grey matter volume (Chen et al., 2012; Tao et al., 2013; Zhang et al., 2012). Although there is a difference between the thalamic volume and grey matter volume of the thalamus, this is actually a discrepancy as grey matter volume of thalamus can be directed related to thalamus volume (Zhang et al., 2012). The study of Montoliu et al. used FreeSurfer (http://surfer.nmr.mgh.harvard.edu) to segment different brain regions and calculate the volume of each brain region, while the other three utilized SPM8 (statistical parametric mapping, http://www.fil.ion.ucl.ac.uk/spm) to generate the grey matter volume images. Thus, these inconsistent results are most likely software dependent.

On the other hand, hepatic encephalopathy (HE) is a metabolic neuropsychological syndrome mainly developed from chronic liver disease which usually leads to liver cirrhosis. Altered brain structure in cirrhotic patients has already been reported in many studies (Chen et al., 2012; Garcia-Garcia et al., 2017; Zhang et al., 2012). Most of these studies focused on the grey matter change in the cortex and striatum, while only one study investigates the relationship between structural changes in thalamus and cirrhotic symptoms (Tao et al., 2013). Thus, the relationship of thalamus structural change and cirrhotic symptoms has not yet been fully elucidated.

To overcome these two issues, we manually measured the volume of thalamus as gold standard and repeated two kinds of VBM analyses in SPM and FreeSurfer, separately. We seek to determine the exact volume change of the thalamus and evaluate the accuracy of the two automatic methods. After that, we correlate the thalamic volumetric measurements to blood biochemical indexes and performance of neuropsychological tests in cirrhotic patients with and without MHE to further investigate the relationship between structural changes in thalamus and liver-brain Abnormality.

## 2 Materials and methods

### 2.1 Participants

This study was approved by the Institutional Ethics Committee of Zhongda Hospital, Medical School of Southeast University (Nanjing, China), with signed informed consent obtained from all the participants.

A total of 33 cirrhotic patients with current MHE (MHE group), 45 cirrhotic patients without MHE (No-HE group), and 21 healthy controls (Control group) were enrolled in this study from 2011 to 2017. Firstly, patients who were diagnosed as hepatitis B associated cirrhosis at the time were enrolled from the community. According to the performance of the neuropsychiatric assessments described below, the cirrhotic patients were either classified as patients with current MHE or patients without MHE. Exclusion criteria for cirrhotic patients were overt HE or a history of overt HE, liver cancer, other neuropsychiatric diseases, history of brain surgery, alcohol or drug abuse, history of recent use of drugs affecting cognitive functions (<2 months), and contraindications to MR examinations. The healthy controls were also enrolled from the community and matched to the cirrhotic patients in terms of age, gender, and education levels.

### 2.2 Laboratory tests

Blood biochemical examinations were performed on 21 patients of MHE group and 33 of No-HE group within one week before MR scanning. Biochemical indexes related to liver cirrhosis, including total bilirubin levels, albumin levels, ALT levels, AST levels, ratio of AST and ALT, and blood coagulation time, were collected. The Child-Pugh scores of these patients were also assessed by combining blood biochemical examinations and MR examinations.

### 2.3 Neuropsychological assessments

The performances of neuropsychological tests, consisting of Number Connection Test A, Number Connection Test B, Digit Symbol Test, and Block Design Test, were evaluated in all the subjects an hour before MR examinations. Cirrhotic patients who showed no overt symptoms of HE but with scores of at least one of the four tests beyond 2 SD (standard deviation) of the mean value for the age-matched controls were defined as MHE patients (14).

### 2.4 MR imaging data acquisition

MR imaging data were acquired with a 1.5 T MRI scanner (Vantage Atlas; Toshiba, Nasu, Japan). A T1-weighted three-dimensional spoiled gradient-recalled sequence was used to acquire the structural images using the following parameters: sagittal; field of view, 250 × 250 mm; in-plane resolution, 256 × 256; 108 slices; slice thickness, 1.5 mm; repetition time, 1200 ms; echo time, 5 ms; inversion time, 500 ms; and section gap, 0 mm.

### 2.5 Image data processing

The DARTEL method in SPM8 (statistical parametric mapping, http://www.fil.ion.ucl.ac.uk/spm) package was used for the segmentation and normalization of the T1 structural images. Structural images of head were firstly checked for sample homogeneity to eliminate poor quality images, and then interactive reorientation was applied. After that, the bias-corrected images were segmented into grey matter, white matter, and cerebrospinal fluid and then normalized to standard Montreal Neurological Institute (MNI) space. Both the modulated grey matter images (grey matter volume/amount) and non-modulated grey matter images (grey matter density/concentration) were smoothed with a Gaussian kernel of 8 mm full width at half maximum (FWHM) and selected for the statistical analysis.

In FreeSurfer (v6.0.0, http://surfer.nmr.mgh.harvard.edu), the semi-automated cortical reconstruction was performed in all the subjects (Montoliu et al., 2012). The volume of the following subcortical regions were extracted: cerebellum, thalamus, caudate, putamen, globus pallidus, hippocampus, amygdala, accumbens, brainstem, and corpus callosum.

For manually measured thalamic volume, the ROIs were manually drawn by two radiologists, one with 5 years of experience and the other with 3, respectively. The thalamus was delineated on the T1 image using the “Autoclose pen” tool in MRIcron.

### 2.6 Statistical analysis

The intra-observer (measure twice for each reader) and inter-observer (Reader 1 vs. Reader 2) reproducibility evaluations of the manually measured thalamic volume were analyzed in SPSS version 18.0 using a two-way mixed model (SPSS Inc. Chicago, IL, USA).

A voxel wise ANOVA analysis of the grey matter volume difference across the whole brain was firstly performed using the smoothed grey matter volume images of the three groups in REST toolkit (REST 1.8, http://www.restfmri.net), with whole brain grey matter volume as covariate. Multiple comparison corrections were performed using family wise error rate correction with a cluster defining threshold *P =* 0.001 (two tail), corresponding to a cluster level of *P =* 0.05 (Eklund et al., 2016). Post-hoc *t*-tests with Bonferroni correction were used to compare the intergroup differences within the mask of the ANOVA results. Whole brain grey matter (GM) and white matter (WM) volume were also calculated and compared among three groups.

A ROI based analysis of average grey matter volume of the thalamus was then performed after extracting the grey matter volume using the mask of thalamus in the AAL (Automated Anatomical Labeling) atlas. The grey matter volume of other brain regions, which exhibit significant differences in the grey matter volume in the voxel-wise ANOVA analysis, were also extracted for further analysis. The differences in the average thalamic grey matter volume, thalamic volume measured automatically and manually, scores of neuropsychological tests, and biochemical data were analyzed using the SPSS software. A one-way ANOVA was hired to detect significant differences in continuous variables such as demographic data, neuropsychological data, and VBM data, while a Chi-square test was used to detect gender significance among groups. A Fisher's least significant difference (LSD) test was used to perform post-hoc multiple comparisons. Two Sample *t*-test and Mann-Whitney U test were used to detect the significant differences in blood biochemical data between MHE and No-HE groups for normally distributed variables and abnormal distributed variables, respectively. *P* values <0.05 were regarded as statistically significant.

The Pearson correlations were performed in SPSS between the manually measured thalamic volume and average grey matter volume of the thalamus as well as thalamic volume measured by FreeSurfer. Then, Pearson correlation was again utilized to evaluate the association between the thalamic volume related parameters and blood biochemical data or neuropsychological test scores. Multiple correlations were corrected with a Bonferroni correction method (*P* ≤ 0.05/30).

## 3 Results

### 3.1 Demographic and clinical profiles

The demographic and clinical data are summarized in Table 1. There were no significant differences in age, gender, or education levels among the three groups. The patients of MHE group had the poorest performance in the neuropsychological tests among the three groups, while the patients of No-HE group basically showed a performance comparable to the healthy controls.

**Table 1:** Demographic and clinical variables of the three groups.

Variable	MHE (n = 33)	No-HE (n = 45)	Controls (n = 21)	*P*
Age	50.88 ± 8.44	49.93 ± 8.19	50.58 ± 8.07	0.747
Gender (M/F)	27/6	38/7	17/4	0.924
Years of education	7.72 ± 2.55	8.44 ± 3.03	8.33 ± 2.44	0.508
Neuropsychological tests	MHE(n = 21)	No-HE(n = 33)	controls(n = 21)	
NCT-A score	75.29 ± 19.30	46.30 ± 18.74	47.35 ± 14.41	<0.001
NCT-B score	176.14 ± 45.93	125.91 ± 46.30	111.15 ± 27.60	<0.001
DST score	25.24 ± 7.62	41.15 ± 11.00	43.33 ± 9.12	<0.001
BDT score	18.24 ± 8.24	29.48 ± 9.80	31.20 ± 7.89	<0.001
Clinical profile	MHE(n = 21)	No-HE(n = 33)		
Total bilirubin levels	112.39 ± 172.49	25.08 ± 15.98	N/A	0.031
Albumin levels	35.18 ± 7.16	38.18 ± 7.96	N/A	0.157
AST levels	38.40 ± 21.53	42.81 ± 23.09	N/A	0.485
ALT levels	57.52 ± 45.42	49.75 ± 27.46	N/A	0.436
AST/ALT	1.62 ± 0.94	1.29 ± 0.53	N/A	0.107
Blood coagulation time	21.93 ± 7.15	17.92 ± 4.03	N/A	0.011
Child-Pugh Score	7.66 ± 2.20	7.09 ± 1.97	N/A	0.322
Child Pugh A/B/C	9/7/5	16/13/4	N/A	0.531

Values are expressed as mean ± SE. Cirrhotic patients with minimal hepatic encephalopathy (MHE), Cirrhotic patients without minimal hepatic encephalopathy (No-HE). NCT-A, Number Connection Test A. NCT—B, Number Connection Test B. DST, Digit Symbol Test. BDT, Block Design Test. AST, aspartate aminotransferase. ALT, alanine transaminase.

### 3.2 VBM analysis in SPM

**Fig. 1:**
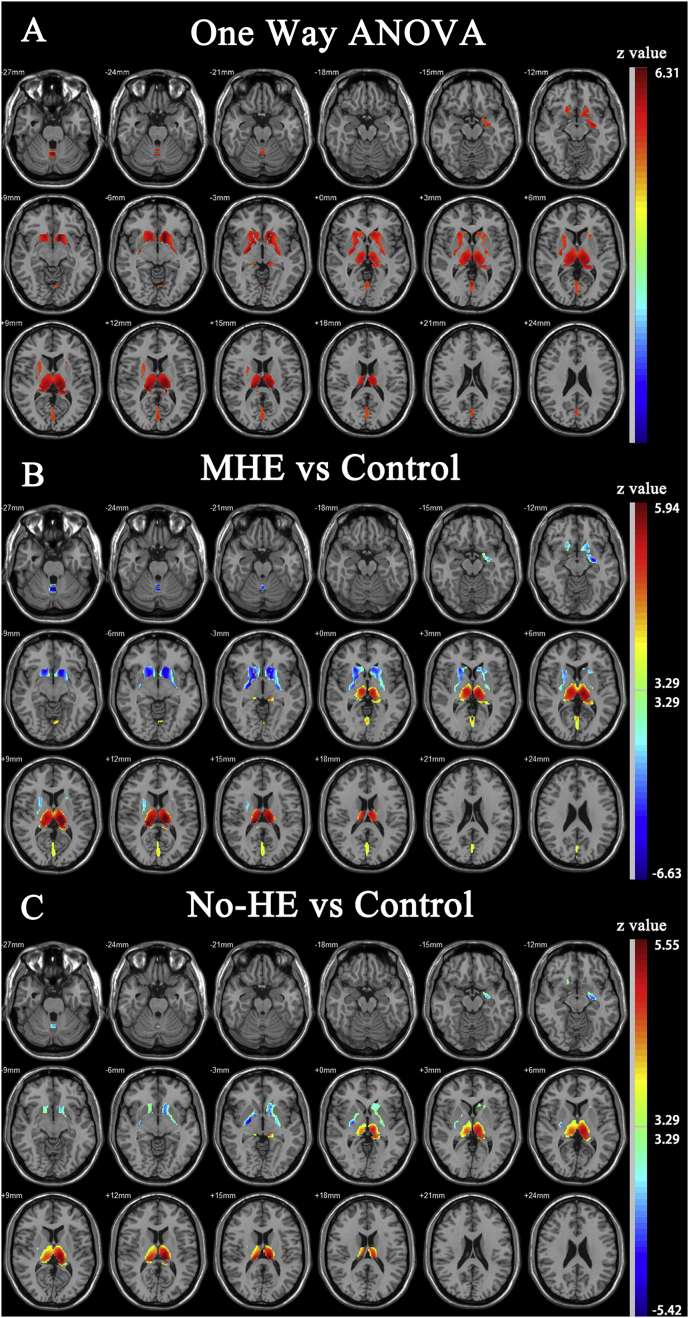
One way ANOVA analysis of grey matter volume among three groups. A, brain area showing significant difference in grey matter volume among three groups (Red). B, Compared with healthy controls, MHE group exhibited decreased grey matter volume in bilateral caudatum and globus pallidus, left hippocampus, and cerebellar vermis (Blue). Significant increased grey matter volume was also observed in bilateral thalamus and the calcarine cortex (Red). C, Similar findings also showed in No-HE group, but the areas showing significance was confined.

**Fig. 2:**
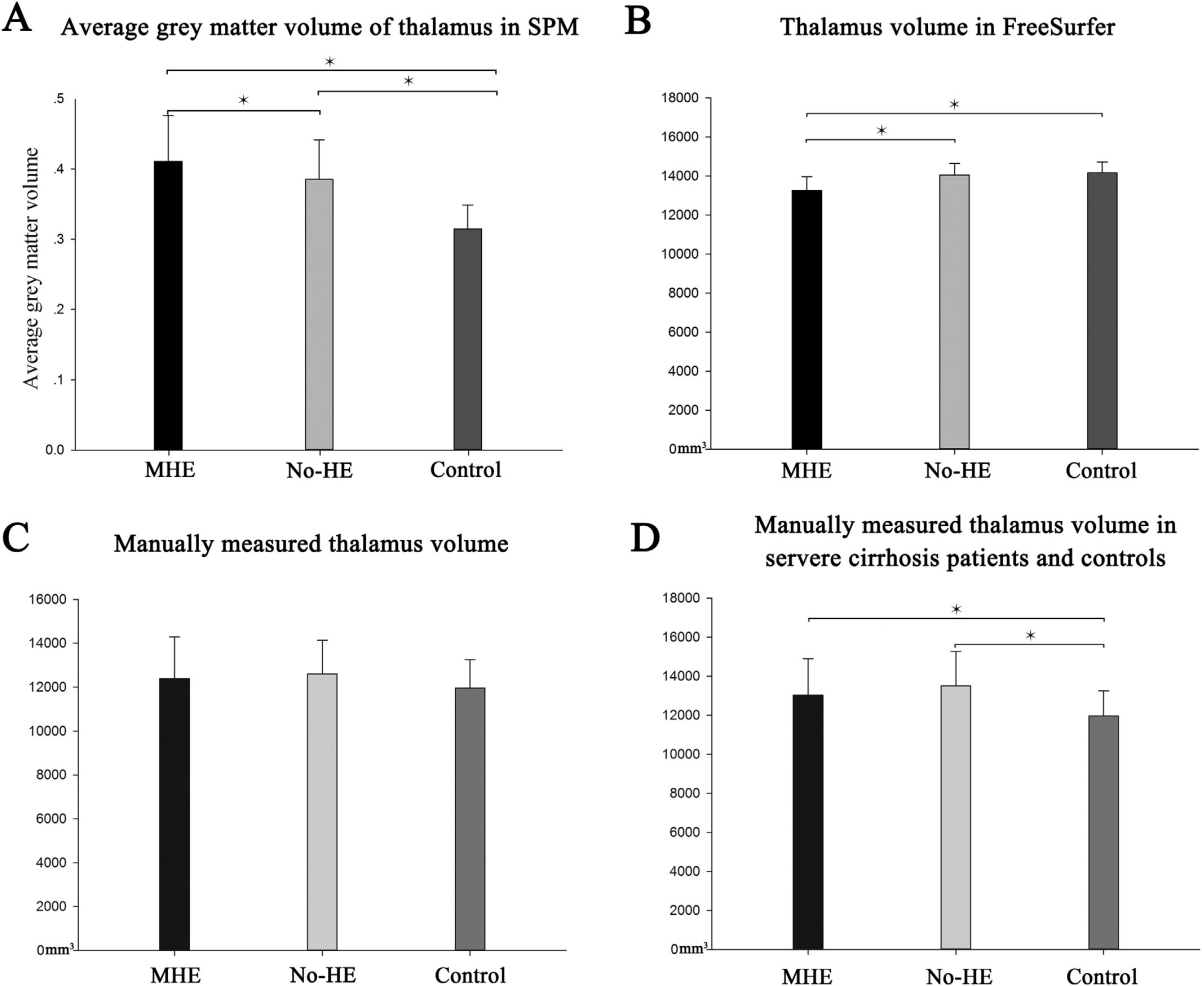
Volumetric measurements of thalamus. A, Both MHE group and No-He group exhibited a higher average thalamic grey matter volume than healthy controls. The MHE group also had a higher average thalamic grey matter volume than No-HE group. B, Both MHE group and No-He group exhibited a lower thalamic volume estimated by FreeSurfer than healthy controls. C, Mean thalamic volume manually measured by a radiologist in three groups. D, When cirrhotic patients who ranked class B and class C in Child-Pugh score only were included, both MHE group and No-HE group had a larger thalamic volume than control group.

Decreased grey matter volume was found in bilateral caudatum and globus pallidus, left hippocampus, and cerebellar vermis in cirrhotic patients, while significant increased grey matter volume was observed in bilateral thalamus and the calcarine cortex (Fig. 1). The grey matter change in the thalamus ranked the highest difference level (highest peak F value in all the clusters) of the A NOVA analysis. The ROI analysis showed the cirrhotic patients exhibited a higher average grey matter volume in thalamus than healthy controls (F = 19.569, *P* < 0.0001, Fig. 2A). The patients of MHE group also had a higher thalamic grey matter volume than the No-HE group in the post-hoc analysis (*P =* 0.049). There was no difference in whole brain grey matter (GM) and white matter (WM) volumes among three groups (Table 2).

**Table 2:** Whole brain grey matter (GM) and white matter (WM) volume.

	MHE	No-HE	Control	F	*P*
Total GM volume	0.70 ± 0.05	0.70 ± 0.05	0.70 ± 0.05	0.026	0.974
Total WM volume	0.51 ± 0.04	0.51 ± 0.04	0.52 ± 0.05	0.716	0.491
Brain Parenchyma	1.21 ± 0.09	1.21 ± 0.09	1.22 ± 0.10	0.097	0.908

Note: Values are expressed as mean ± SE, the unit is liter (L). GM, grey matter; WM, white matter; One way ANOVA analysis was performed to detect the difference of GM and WM volume among three groups.

### 3.3 Thalamic volume measurements in FreeSurfer

Volumes of subcortical regions estimated from the volume estimation methods in FreeSurfer were summarized on Table 3. Contrary to the thalamic grey matter volume measurements of SPM, the volume of thalamus is deceased in cirrhotic patients compared to healthy controls (Fig. 2B). The MHE group had the smallest thalamic volume when compared to the No-HE group and control group (*P =* 0.032 and *P =* 0.045, respectively).

**Table 3:** Volume of subcortical nuclei estimated by FreeSurfer for each group.

	Control	No-HE	MHE
Thalamus	14.2 ± 0.5	14 ± 0.5	13.2 ± 0.6⁎,#
Hippocampus	8.6 ± 0.3	8.4 ± 0.3	8.4 ± 0.3
Amygdala	2.9 ± 0.2	2.8 ± 0.2	2.7 ± 0.2
Caudate	7.4 ± 0.2	6.8 ± 0.3⁎	6.7 ± 0.3⁎
Putamen	11.3 ± 0.5	10.3 ± 0.6⁎	10.3 ± 0.6⁎
Globus pallidus	3.5 ± 0.2	3.3 ± 0.3	3.3 ± 0.3
Accumbens	1.2 ± 0.1	1.1 ± 0.1⁎	1.0 ± 0.2⁎

Volumes (cm^3^) are expressed as mean ± SEM. An ANOVA analysis of volumes is preformed among three groups. Fisher's least significant difference (LSD) test was used to do post-hoc multiple comparisons.

⁎ Significant differences between controls and cirrhotic group (*P* < 0.05).

# Significant differences between MHE and No-HE group (*P* < 0.05).

### 3.4 Manual measurements of thalamic volume

Satisfactory inter- and intra-observer reproducibility of manually measured thalamic volume was achieved (Table 4). The first measurement with a lower standard deviation measured by a radiologist (Reader 1) who had a higher intra-observer ICC (intra-class correlation coefficient) was adopted for the analysis of the remaining steps. The ANOVA analysis of the manually measured thalamic volume showed no significant differences among three groups (Fig. 2C, F = 1.132, *P =* 0.327). Although a statistical significance was not reached, the mean of the manually measured thalamic volume appeared to be higher in cirrhotic groups than that of control group.

**Table 4:** Inter- and intra-observer reproducibility of manually measured thalamus volume.

	Intra-observer ICCs	Inter-observer ICCs
Reader 1	0.913 (0.871–0.942)	N/A
Reader 2	0.857 (0.787–0.904)	N/A
Trial 1	N/A	0.880 (0.821–0.919)
Trial 2	N/A	0.886 (0.831–0.924)

ICC, intraclass correlation coefficient; CI, confidence interval.

To further investigate the relationship between the thalamic volume and clinical manifestation of liver cirrhosis, we selected cirrhotic patients who ranked B or C by Child-Pugh score and compared their thalamic volumes to the control group. Unsurprisingly, the cirrhotic patients who had severe liver cirrhosis (ranked B and C in the Child-Pugh score) exhibit a higher thalamic volume than that of control groups (Fig. 2D, *P =* 0.047 for MHE group and *P =* 0.011 for No-HE group, compared to control group).

### 3.5 Correlation analysis

**Fig. 3:**
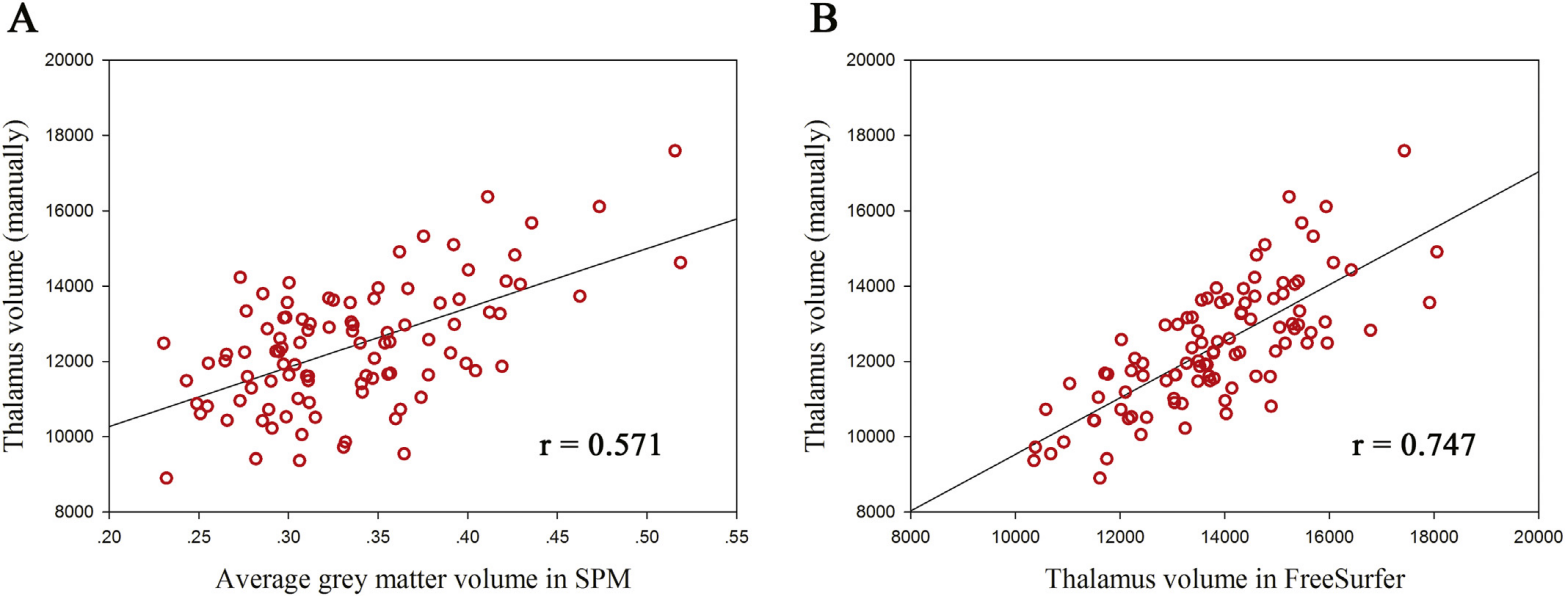
Correlation between automatic measurements and manual measurements. A, Correlation between manually measured thalamus volume and average grey matter volume estimated by SPM. B, Correlation between manually measured thalamus volume and thalamus volume estimated by FreeSurfer.

Firstly, we correlated the manually measured thalamic volume with the average grey matter volume of the thalamus estimated by SPM as well as thalamic volume by FreeSurfer. Both of the two automatic measurements were highly correlated with the manual measurements, with the correlation coefficient of 0.571 (0.396 to 0.712, 95% CI) for SPM and 0.747 (0.668 to 0.819, 95% CI) for FreeSurfer (Fig. 3). The correlation coefficient of the correlation analysis for FreeSurfer was higher than that for SPM, but it did not reach statistical significance as the overlap of the 95% confidence intervals.

**Fig. 4:**
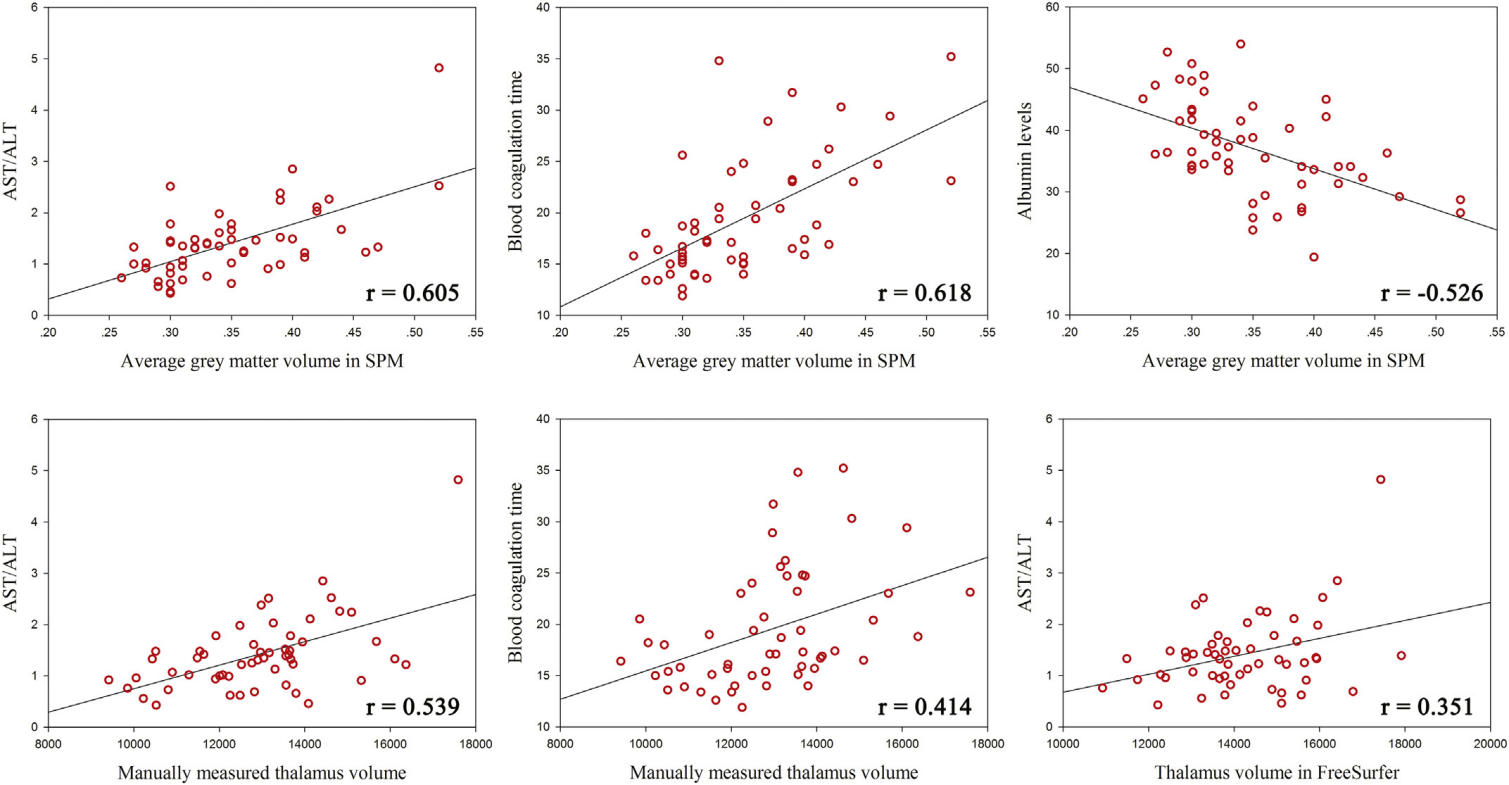
Correlation between thalamic volumetric measurements and blood biochemical indexes. The average grey matter volume of the thalamus closely correlated with AST/ALT ratio, blood coagulation time and albumin levels. Thalamus volume also correlated with AST/ALT ratio and blood coagulation time, though the correlation coefficients decreased considerably.

We then correlated the neuropsychological test scores and blood biochemical indexes related to cirrhosis with the three volumetric measurements of the thalamus (both automatic measurements and manual measurements). The correlation coefficients between all of the each pairs were listed on Table 5. In the Pearson correlation analysis, the average grey matter volume of the thalamus in SPM positively correlated with the AST/ALT ratio (r = 0.605, *P* < 0.0001) and blood coagulation time (r = 0.618, *P* < 0.0001) and negatively correlated with the albumin levels (r = −0.526, *P* < 0.0001) in the cirrhotic patients (Fig. 4). The average grey matter volume of the thalamus also correlated with the Child-Pugh score in the Spearman analysis (r = 0.462, *P* < 0.001). Moreover, the average grey matter volume of the thalamus correlated with the biochemical indexes in the MHE group and No-HE group separately. It correlated with AST/ALT ratio, blood coagulation time, and albumin levels in both the MHE (r = 0.657, *P =* 0.001; r = 0.633, *P =* 0.001; r = −0.555, *P =* 0.001 for AST/ALT, blood coagulation time, and albumin levels, respectively) and No-HE groups (r = 0.497, *P =* 0.003; r = 0.578, *P =* 0.006; r = −0.448, *P =* 0.042 for AST/ALT, blood coagulation time, and albumin levels, respectively). No correlation between average grey matter volume of the thalamus and the neuropsychological tests performance was detected. There also did not exist a correlation between average grey matter volume of other brain regions that showed significance in the voxel wise ANOVA analysis and biochemical indexes as well as neuropsychological test performances.

**Table 5:** Correlation coefficients between thalamic volumetric measurements and clinical indexes.

	NCT_A	DST	NCT_B	BDT	TBL	ALB	ALT	AST	AST/ALT	BT
GM_Volume_SPM	0.006	−0.021	0.020	0.037	0.167	−0.526#	−0.120	0.278	0.605#	0.618#
Volume_FreeSurfer	−0.374	0.247	−0.166	0.266	0.186	0.001	−0.120	0.021	0.351⁎	0.322
Volume_Manual	−0.232	0.137	−0.022	0.115	0.203	−0.312	−0.089	0.203	0.539#	0.414⁎

Correlation coefficients between thalamic volumetric measurements and blood biochemical indexes as well as neuropsychological test performance. GM_Volume_SPM, average thalamic grey matter volume estimated by SPM; Volume_FreeSurfer, thalamic volume estimated by FreeSufer; Volume_Manual, manually measured thalamic volume. NCT-A, Number Connection Test A; NCT—B, Number Connection Test B; DST, Digit Symbol Test; BDT, Block Design Test; TBL, total bilirubin levels; ALB, albumin levels; AST, aspartate aminotransferase; ALT, alanine transaminase; BT, blood coagulation time.

⁎ *P* < 0.01.

# *P* < 0.0001.

Finally, we tried to determine whether the thalamic volume also directly correlated with these parameters mentioned above. The correlation analysis showed that the manually measured thalamic volume was associated with the AST/ALT ratio (r = 0.539, *P* < 0.001) and blood coagulation time (r = 0.414, *P =* 0.002). There also exists a correlation between the thalamic volume measured in FreeSurfer and AST/ALT ratio (r = 0.351, *P =* 0.009). No correlation was found between the thalamic volume measured by these two methods and neuropsychological test performance.

## 4 Discussion

The brain areas of altered grey matter volume detected by VBM analysis in SPM are coincident with previous studies (Chen et al., 2012; Montoliu et al., 2012; Tao et al., 2013; Zhang et al., 2012). Symmetrical grey matter alterations appeared in the basal ganglia nucleus and thalamus of cirrhotic patients, with the alteration being greater and more extensive in MHE group than No-HE group. The ROI analysis showed a higher average thalamic grey matter volume in cirrhotic patients than healthy controls. Meanwhile, the volume estimation of subcortical regions in FreeSurfer is also consistent with the study of Montoliu et al.'s. Thus, the inconsistent results of thalamic structural change were successfully reproduced.

The manually measured thalamic volume revealed that the thalamic volume in cirrhotic patients was increased rather than decreased and reached statistical significance in severe cirrhotic patients with hepatic decompensation compared to healthy controls. Hence, the manually measured results confirmed that cirrhotic patients had a larger thalamic volume, which is contrary to the results of FreeSurfer and resembled the results of SPM. It is beyond the scope of the present study to find out the reason why FreeSurfer yield a result opposite to the manual method. However, a careful interpretation is needed for the increased thalamic volume in cirrhotic patients. Zhang et al. indicates neuronal and/or glial hypertrophy or hyperplasia may be the reason for increased thalamic volume (Zhang et al., 2012). Yet, there is still no direct evidence supporting this assumption. Meanwhile, increased grey matter volume observed on the MR image can also be caused by neurogenesis, extensive training and brain oedema (Abela et al., 2015; Draganski et al., 2004; Freund et al., 2012; Kuhn et al., 2014; Zivadinov et al., 2008). We have begun to investigate this apparent structural change using rodent models. On the other hand, the measurement of SPM seems to be more accurate than that of FreeSurfer for inter-group comparison; however, the thalamic volume estimated by FreeSurfer outperforms the thalamic grey matter volume in the correlation analysis with manually measured thalamic volume. Firstly, the segmentation of the brain in SPM is implemented with a tissue classification step and a registration step (Ashburner and Friston, 2005). In the tissue classification step, each voxel in the brain of a subject is assigned with probabilities to be grey matter, white matter, and CSF. The probability of grey matter refers to the grey matter density or concentration within each voxel, with a value less than one (100%). Therefore, although the thalamus is a large mass of grey matter in anatomy, it can be considered to be comprised of voxels containing both grey matter and white matter in the MR image from the methodological view of brain tissue segmentation algorithm implemented in the SPM. Thus, the grey matter volume of thalamus is not equal to the thalamic volume and always has a smaller value than thalamic volume. In fact, the grey matter volume merely involves a modulation or scaling step (Keller et al., 2004), in which the grey matter density/concentration is adjusted by multiplying its relative volume before and after warping. After the modulation, the grey matter volume can be greater than one (100%) in a voxel since the original thalamus could be compressed or contracted during spatial normalization. Intuitively, the correlation between thalamic grey matter volume and volume of thalamus will result in a smaller correlation coefficient than that between two original volume measures. Furthermore, the considerable difference in correlation coefficient can also stem from the measuring error of manually measured thalamic volume. The correlation coefficients between the second measurements of thalamic volume and the measurement of SPM or FreeSurfer were 0.640 or 0.648, respectively, performed by the same radiologist.

The grey matter volume of the thalamus closely correlated with the blood biochemical indexes related to liver cirrhosis. The correlation coefficients remain high even in separate groups. These blood biochemical indexes were not associated with the grey matter volume of other brain regions which also exhibited significant grey matter volume change in the voxel-wise ANOVA analysis. This result indicates the specificity of thalamic structural change in the development of liver cirrhosis. Although a significant correlation can also be observed between these blood biochemical indexes and thalamic volume (both manual and automatic measurements), the correlation coefficients decreased considerably.

Above all, we found that the thalamic volume did not show a significant difference among the three groups and only weakly correlated with the blood biochemical indexes related to the liver cirrhosis, whereas the grey matter volume/amount has a significant difference among the three groups. Given that, it can be concluded that it is the grey matter density that has a significant difference among the three groups since the average grey matter density reflects the concentration or ratio of the grey matter in the thalamus. Actually, the result of grey matter density showed a similar pattern in the voxel-wise analysis and ROI analysis (unpublished data). The correlation analysis also yielded high correlation coefficients between average thalamic grey matter density and blood biochemical indexes (r = 0.408, *P* = 0.002; r = 0.547, *P* < 0.0001; and r = −0.601, *P* < 0.0001 for AST/ALT, blood coagulation time, and albumin levels, respectively). This result indicates that the relative intensity change in the thalamus is more sensitive than the volume change for discriminating cirrhotic patients and healthy controls as well as degree of cirrhosis.

## 5 Conclusion

In conclusion, this study helped to clarify the thalamic volume change in the cirrhotic patients with and without the MHE and found an close association between thalamic structural change and blood biochemical indexes reflecting the degree of cirrhosis. Despite the grey matter change in the cortex and striatum, the grey matter change in the thalamus may also play a critical role in encephalopathy of cirrhotic patients and needs more attention. Finally, this study also provides a good demonstration of the usage and underlying principle of different VBM measurements, which improves the understanding of the VBM method for clinicians.

## Contributors

S.H.J. is the guarantor of this work and has full access to all the data in the study and takes responsibility for the integrity of the data and the accuracy of the data analysis. S.H.J. and Y.J. contributed to the design of the study and revised the manuscript for intellectual content. C.Q.L. collected the data, performed the analysis, and wrote the manuscript. Y.J. and X.P.M. helped to collect the data and contributed to the revision of the manuscript. Y.C., Y.L., and X.M.X. contributed to the analysis of the data.

C.Q.L. and Y.J. contributed equally to this work.

## Declaration of interests

C.Q.L. has nothing to disclose. Y.J. has nothing to disclose. X.P.M. has nothing to disclose. Y.C. has nothing to disclose. Y.L. has nothing to disclose. X.M.X. has nothing to disclose. S.H.J. has nothing to disclose.
